# Identification of the Hub Genes Related to Nerve Injury-Induced Neuropathic Pain

**DOI:** 10.3389/fnins.2020.00488

**Published:** 2020-05-20

**Authors:** Kai Wang, Duan Yi, Zhuoyin Yu, Bin Zhu, Shuiqing Li, Xiaoguang Liu

**Affiliations:** ^1^Department of Pain Medicine Center, Peking University Third Hospital, Beijing, China; ^2^Department of Anesthesiology, Peking University Third Hospital, Beijing, China; ^3^Department of Orthopedic, Peking University Third Hospital, Beijing, China

**Keywords:** neuropathic pain, nerve injury model, bioinformatics analysis, hub gene, functional association network

## Abstract

**Background:**

The reactivity enhancement of pain sensitive neurons in the nervous system is a feature of the pathogenesis for neuropathic pain (NP), yet the underlying mechanisms need to be fully understood. In this study, we made an attempt to clarify the NP-related hub genes and signaling pathways so as to provide effective diagnostic and therapeutic methods toward NP.

**Methods:**

Microarray expression profile GSE30691 including the mRNA-seq data of the spared nerve injury (SNI)-induced NP rats was accessed from the GEO database. Then, genes associated with NP development were screened using differential analysis along with random walk with restart (RWR). GO annotation and KEGG pathway analyses were performed to explore the biological functions and signaling pathways where the genes were activated. Afterward, protein-protein interaction (PPI) analysis and GO analysis were conducted to further identify the hub genes which showed an intimate correlation with NP development.

**Results:**

Totally 94 genes associated with NP development were screened by differential analysis and RWR analysis, and they were observed to be predominantly enriched in hormone secretion and transport, cAMP signaling pathway and other NP occurrence associated functions and pathways. Thereafter, the 94 genes were subjected to PPI analysis to find the genes much more associated with NP and a functional module composed of 48 genes were obtained. 8 hub genes including C3, C1qb, Ccl2, Cxcl13, Timp1, Fcgr2b, Gal, and Lyz2 were eventually identified after further association and functional enrichment analyses, and the expression of these 8 genes were all higher in SNI rats by comparison with those in Sham rats.

**Conclusion:**

Based on the data collected from GEO database, this study discovered 8 hub genes that were closely related to NP occurrence and development, which help to provide potent theoretical basis for NP treatment.

## Highlight

– 94 genes closely related to neuropathic pain occurrence are identified using differential analysis and random walk with restart.
– 8 hub genes that are implicated with neuropathic pain regulation are verified by means of protein association analysis along with GO annotation and KEGG pathway analyses.

## Introduction

Pain is a survival mechanism that can act as a warning sign of ongoing or impending tissue damage. In evolutionary terms, the activation of high threshold mechanical nociceptors or other types of specialized nociceptor plays a protective role and can serve as a warning system for dangerous stimuli (Cohen and Mao, 2014). Neuropathic pain (NP) is a kind of chronic pain induced by the injury or dysfunction of the central or peripheral nervous system (Jensen et al., 2011; Finnerup et al., 2016; Watson and Sandroni, 2016). Smith et al. (2007) discovered that compared with the nociceptive pain, NP produced a more negative impact on the life quality. However, the specific mechanisms underlying NP remain elusive and there is still a lack of the effective therapeutic methods. Therefore, it is urgent to further clarify the underlying mechanisms toward NP and exploit the relevant genes and signaling pathways, so as to provide theoretical basis and new ideas for future treatment.

The pathogenesis of NP is complex. The current discovery has shown that NP is not only involved in the excitability of transmitting pain sensitive neurons, but also related to central and peripheral sensitization (von Hehn et al., 2012; Meacham et al., 2017). Central pain, a subtype of NP (like spinal cord injury-induced pain), manifests as a series of symptoms and signs that are developed after the injury of the central nervous system, such as nerve pain caused by headache, abdominal pain, etc. (Cohen and Mao, 2014). In addition, other than the inducement of inflammatory response in some local tissues, peripheral nerve injury or tissue damage can also cause alterations of the inflammatory-related cytokines in the central nervous system, such as the elevation of interleukin-1β (IL-1β), IL-6, tumor necrosis factor-α (TNF-α), chemokines and neurotrophic factors (Rubio and Sanz-Rodriguez, 2007; Wei et al., 2012; Matsuo et al., 2014; Sato et al., 2014; Gerard et al., 2015). Zhang et al. (2013) reported that inhibiting CXCL1-CXCL2 signal might be used as a novel therapeutic method for NP treatment. Moreover, Xiong et al. (2016) found that M1-type small glial cells could produce a large number of pro-inflammatory factors, resulting in the aggravation of nerve injury and consequently leading to the neurological dysfunction. Hence, further investigating the molecular mechanisms underlying NP and clarifying the effective targets are significant for the application of pain medication in clinical targeted therapies.

Bioinformatics can provide tools for analysis of large amounts of information, like the microarray technique, which has been widely applied in high-throughput gene expression detection (Schena et al., 1995; Allison et al., 2006) and can be reliably used for the identification of novel targets for clinical diagnosis and treatment (Chen et al., 2015). This study aimed to discuss the molecular mechanisms of nerve injury-induced NP, and in turn identify the hub genes and signaling pathways associated with NP pathogenesis. Due to the certain difficulties and the risk of experimenting on human being, we adopted animal models to study the NP pathogenesis. In our study, microarray GSE30691 including the mRNA-seq data of the spared nerve injury (SNI)-induced NP rats was downloaded from the GEO database. Multiple bioinformatics methods were adopted here for screening the genes and pathways which were associated with NP occurrence. In the meantime, hub genes intimately relevant to NP development were identified. Our findings would provide new thoughts for exploration of genes and biological pathways that are involved in nerve injury-induced NP.

## Materials and Methods

### Data Source

The mRNA expression microarray GSE30691 was downloaded from the Gene Expression Omnibus (GEO) database^1^. The dataset was composed of the L4-5 dorsal root ganglion (DRG) segments from the rats at 0, 3, 7, 21, and 40 days after SNI and from the rats at 3, 7, and 21 days after a sham operation. Three independent experiments were performed in each period.

### Differential Analysis

Statistical software R (version 3.3.2)^2^ and packages of Bioconductor^3^ were applied for analysis of the differentially expressed genes (DEGs). Differential analysis was performed on the genes from the SNI and Sham rats in 3, 7, and 21-day three time periods using the “limma” package (Smyth, 2011), with | logFC| > 0.585 and FDR < 0.05 used as the screening threshold.

### Random Walk With Restart (RWR) for Screening NP-Related Genes

In order to make the analysis more reliable, a network which can execute on the protein-protein interaction (PPI) network was designed. RWR is a classic ranking algorithm which is originated from the random walk. With the aid of RWR analysis, the global structure information of the network can be explored, which is helpful to estimate the proximity between two nodes (Zhang et al., 2018; Fan et al., 2019; Valdeolivas et al., 2019).

The PPI network we had constructed was denoted as a graph G = (V, E) comprising of a set of genes V and a set of interactions E. The graph could be characterized by an n × n adjacency matrix A:

(1) $$A_{{\left[i,j\right]}^{\prime }}=\frac{A_{\left[i,j\right]}}{\sum_{k=1}^{n}A_{\left[i,j\right]}}$$

where n refers to the total number of the nodes. A_([i,j]) = 1 if node i and node j are interacted, and 0 otherwise. In the RWR algorithm, each node in the network was conferred a restart probability and all probabilities constituted a vector which was defined as Pt:

(2) $$P\,\_\,\left(t+1\right)=\left(1-r\right)\,A^{\wedge^{\prime }}\,P\,\_\,t+\mathrm{rP}\,\_\,0$$

where A is the column-wise normalized adjacency matrix A. Pt is the previous state probabilities at time t. r is the restart probability. P0 is the initial state probabilities, a column vector with 1/m for the m seed genes (NP-related genes identified in L4-5 DRG segments from SNL cohort) and 0 for other genes on the network.

The iteration process was repeated until the difference between two vectors was smaller than 1 × 10^–5^. New NP-related genes were subsequently identified and Venn diagram was plotted to obtain RWR genes.

### GO Annotation and KEGG Pathway Analyses

As we had screened the genes associated with NP using the RWR analysis, R package “clusterProfiler” was used to perform GO annotation and KEGG pathway analyses, with the critical value of *p* < 0.05 and *q* < 0.05 (Yu et al., 2012). Afterward, the enrichment results were visualized with the aid of R package “enrichplot” so as to further analyze the biological functions and pathways by which the genes affected NP.

### PPI Network Construction

The NP-associated genes we identified were projected onto a PPI network for functional association analysis (confidence > 0.400) using the STRING database^4^. Thereafter, the Cytoscape plugin “MCODE” was applied to find the functional module, while “ClueGO” and “CluePedia” were used for enrichment analysis toward the genes in the module.

## Results

### Identification of NP-Associated Genes

**FIGURE 1:**
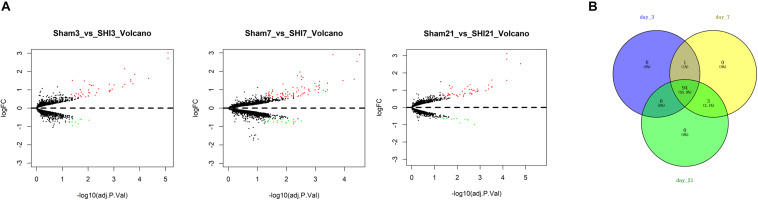
Identification of NP-associated genes. **(A)** Volcano Plots of the DEGs in the SNI group versus the Sham group at 3, 7, and 21 days after surgery, respectively; **(B)** Venn diagram showed the common NP-associated genes from the 3 time periods.

To find the genes that were tightly correlated with NP, differential analysis was run for the genes in the microarray GSE30691. The results revealed that a total of 51, 99, and 63 DEGs were identified from the SNI group versus the Sham group at 3, 7, and 21 days after SNI, respectively (Figure 1A). Thereafter, the DEGs were projected onto a PPI network, and the DEGs of each time period were regarded as seed genes for follow-up RWR analysis. Eventually, a total of 95, 98, and 97 NP-associated genes were screened in three periods, respectively, and the 94 common genes identified using a Venn diagram were considered to be closely correlated with NP (Figure 1B).

### GO and KEGG Analyses on the NP-Associated Genes

**FIGURE 2:**
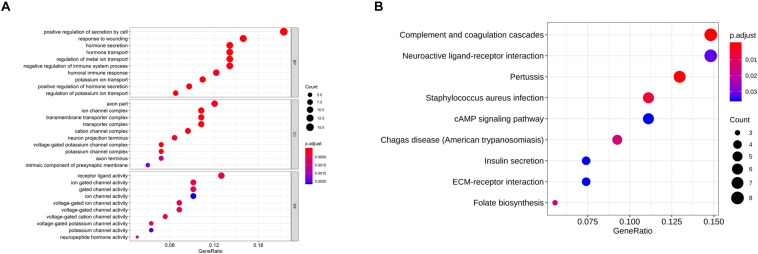
GO and KEGG analyses on the NP-associated genes. **(A)** The most enriched GO terms of the 94 NP-associated genes; **(B)** the most activated KEGG pathways of the 94 NP-associated genes.

As abovementioned, 94 genes were identified to be closely related to NP. In order to investigate the role of these genes in NP, GO annotation and KEGG pathway analyses were conducted. As revealed in Figure 2A, the most significantly activated biological functions of these genes were hormone secretion and transport, potassium ion transport, humoral immune response and negative regulation of immune system process, etc. While the most noteworthy enriched signaling pathways were complement and coagulation cascade, neuroactive ligand-receptor interaction, cAMP signaling pathway and ECM-receptor interaction, etc. (Figure 2B). All of these functions and pathways have been proven to show an intimate correlation with NP development, which supports our result that the 94 genes we identified are significantly associated with NP.

### PPI Network Analysis and Identification of Hub Genes

**FIGURE 3:**
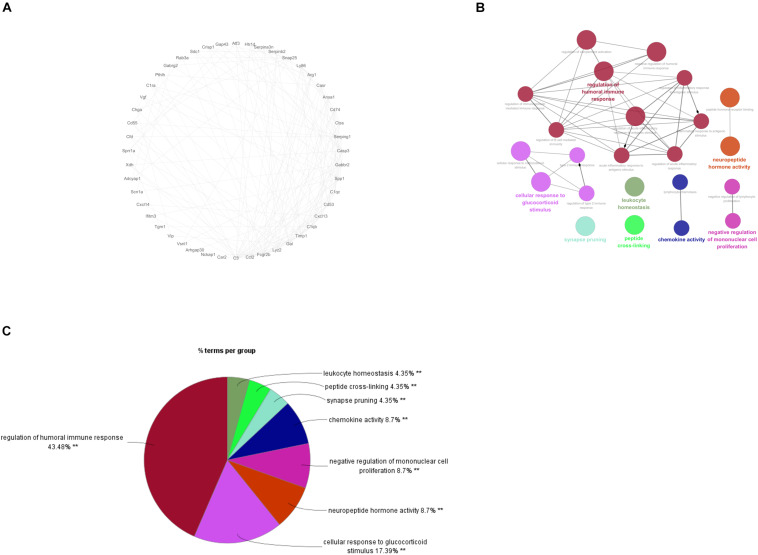
PPI Network Analysis and identification of hub genes. **(A)** PPI network for the 48 genes involved in the functional module of a highest score; **(B)**: the most enriched GO terms for the 48 genes; C: Proportional graph of the enriched GO terms of the 48 genes.

To gain more insight into the role of these 94 genes in NP development and find the hub genes which were significantly implicated in, a PPI network based on these 94 genes was established on STRING database for functional association analysis and sequentially visualized on Cytoscape. The plugin “MCODE” was used to find functional modules and eventually a module consisting of 48 genes with the highest score was obtained (Figure 3A). After that, biological functions where the 48 genes were most activated were explored by means of GO annotation. It turned out that the genes were predominantly enriched in some NP development associated functions, including regulation of humoral immune response, cellular response to glucocorticoid stimulus, neuropeptide hormone activity, negative regulation of mononuclear cell proliferation and chemokine activity (Figures 3B,C).

**FIGURE 4:**
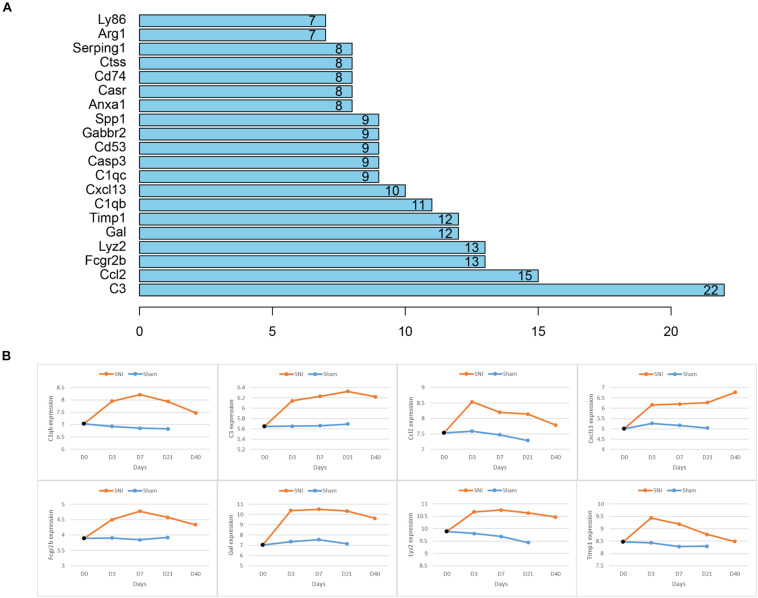
Hub genes associated with NP and the relative expression of each hub gene in different time periods between SNI and Sham rats. **(A)** Node number of the hub genes in the PPI network; **(B)** the relative expression of each hub gene with a degree higher than 10 in different time periods between Sham and SNI rats.

As the 48 genes in the module were found to be enriched in NP-associated functions, further PPI network analysis was conducted to identify the hub genes that were most relevant to NP occurrence and development. The connectivity degree of each gene was calculated and it turned out that 8 genes (C3, C1qb, Ccl2, Cxcl13, Timp1, Fcgr2b, Gal, Lyz2) which had a degree higher than 10 were identified and here were regarded as the hub genes significantly associated with NP development (Figure 4A). For further verification, we detected the expression of the 8 genes in different time periods between SNI and Sham rats and found that all these 8 genes exhibited a much higher expression in SNI rats in comparison with those in Sham rats in the same period (Figure 4B). In view of these results, the 8 hub genes were confirmed to be most associated with NP development.

## Discussion

NP is a complex chronic pain with elusive mechanisms currently (Kerstman et al., 2013; Gilron et al., 2015). It has been reported that NP is commonly associated with paresthesia, hyperesthesia, paralgesia and hyperalgesia (Bouhassira and Attal, 2016). In addition, some changes in the whole nervous system are also implicated with NP, such as the ectopic action potential, the generation of the new synaptic circuitry and the neuro-immune interaction (Taylor, 2001; Zhuo et al., 2011). Therefore, it is a necessity to extend our knowledge on the NP pathogenesis, which is of great significance on setting of the treatment strategies for responsive NP prevention and efficacy improvement.

It has been revealed that NP is always accompanied by the alteration of genes on the sensory pathways (von Hehn et al., 2012). In the present study, we adopted the microarray technique to identify the NP-related DEGs and the activated signaling pathways from the SNI rat models. Microarray technique is a tool able to quantify the expression levels of thousands of genes across the biological samples simultaneously, and it can provide the complex regulations among genes based on the expression data of the whole genome, which helps us find better targets for NP treatment (Gao et al., 2018). During the whole analysis process, some factors like the sample attributes, processing tools, handling methods and results screening all made some effects on the final results. To make the results more reliable, we used multiple analytical methods, such as differential analysis, RWR, GO annotation, and KEGG pathway analyses. More specifically, mRNA expression data from the SNI and Sham rats 3, 7, and 21 days after operation were obtained from microarray GSE30691 through the GEO database. Subsequently, DEGs in the three time periods were screened and projected onto a PPI network, after which the DEGs in each period were taken as seed genes for RWR analysis. Eventually, 94 common genes were identified and considered to be associated with NP development. Our findings lay a foundation for future investigation of the molecular mechanisms underlying NP occurrence and development.

After identification of the NP-associated genes, we performed enrichment analysis and found that these genes were predominantly enriched in some biological functions like hormone secretion and transport, potassium ion transport, humoral immune response, negative regulation of immune system process, while these functions have been proven to be involved in NP occurrence and development (Jaggi et al., 2015; Jang et al., 2018; Wang et al., 2018). Additionally, cAMP signaling pathway and ECM-receptor interaction are two signaling pathways that have been confirmed to be implicated with multiple functions in regulation of NP (Zhou et al., 2017; Yan et al., 2018; Yan et al., 2019), and our study observed that the genes we identified were activated in these two pathways as well. Given the findings above, the specific role of these genes in NP development requires further exploration.

Despite the genes and pathways associated with NP development we found, 8 hub genes (C3, C1qb, Ccl2, Cxcl13, Timp1, Fcgr2b, Gal, Lyz2) that were responsible for NP development regulation were identified and some of them have been reported to present an intimate correlation with NP development. Levin ME et al. conducted the microarray analysis on the data from the SNI-induced NP rats, and the results demonstrated that multiple complement factors like C1 inhibitor, C1q α, β, and γ, C1r, C1s, C2, C3, C4, and C7 were all up-regulated, and rats with less complement C3 in plasma (cobra venom factor-treated) had relative attenuated pain behaviors (Levin et al., 2008). This study found that C3 was remarkably increased in SNI rats and exhibited a rising trend within 0–21 days. As for Timp1, Gal and C1qb, researchers discovered that Gal and C1qb could be used as potential biomarkers for NP occurrence (Buckley et al., 2018; Yang et al., 2018). Besides, a study on CXCL13 made by Jiang et al. (2016) revealed that CXCL13 could make an effect on NP development via targeting CXCR5. These genes were all observed to be significantly highly expressed in SNI rats in our study. Moreover, CCL2 has been verified to play a vital role in NP development (Zhao et al., 2017), yet there has been no study performed to investigate the role of Fcgr2b and Lyz2 in NP. Overall, our identification of the 8 hub genes further confirms their significance in NP development.

In conclusion, we found 94 NP-associated genes and corresponding enriched biological functions and signaling pathways by means of multiple bioinformatics approaches. Furthermore, 8 hub genes that were implicated with NP development regulation were identified. Our findings lay a foundation for future exploration of the molecular mechanisms underlying NP development and help to find potential targets for NP diagnosis and treatment.

## Data Availability Statement

The datasets analyzed in this study were downloaded and accessed from the Gene Expression Omnibus (GEO) database: https://www.ncbi.nlm.nih.gov/geo/, with accession no: GSE30691.

## Author Contributions

KW, DY, ZY, BZ, SL, and XL: study design, wrote the paper, and revised the manuscript and gave the final approval of the version. KW, DY, and ZY: literature search. BZ, SL, and XL: acquired the data.

## Conflict of Interest

The authors declare that the research was conducted in the absence of any commercial or financial relationships that could be construed as a potential conflict of interest.

## Footnotes

1 https://www.ncbi.nlm.nih.gov/geo/

2 https://www.r-project.org/

3 http://www.bioconductor.org/

4 https://string-db.org/cgi/input.pl
